# Editorial: Impact of Breast MRI on Breast Cancer Treatment and Prognosis

**DOI:** 10.3389/fonc.2022.825101

**Published:** 2022-03-11

**Authors:** Almir Bitencourt, Mami Iima, Georg Langs, Katja Pinker

**Affiliations:** ^1^Imaging Department, A.C.Camargo Cancer Center, São Paulo, Brazil; ^2^Breast Imaging Section, DASA, São Paulo, Brazil; ^3^Department of Diagnostic Imaging and Nuclear Medicine, Kyoto University Graduate School of Medicine, Kyoto, Japan; ^4^Institute for Advancement of Clinical and Translational Science (iACT), Kyoto University Hospital, Kyoto, Japan; ^5^Department of Biomedical Imaging and Image-guided Therapy, Medical University of Vienna, Vienna, Austria; ^6^Department of Radiology, Breast Imaging Service, Memorial Sloan Kettering Cancer Center, New York, NY, United States

**Keywords:** breast cancer, magnetic resonance imaging, artificial intelligence, radiomics, prognosis, treatment

Breast cancer treatment and prognosis depends on the extent of the disease at diagnosis (staging) and biological characteristics of the tumor. While staging is mainly based on clinical and imaging features, biological characteristics used to define treatment are usually based on the analysis of needle biopsy samples, which represent only a small portion of the tumor and are subject to sampling bias (1, 2). Breast Magnetic Resonance Imaging (MRI) has become a fundamental method in the management of breast cancer patients. In addition to morphology assessment, MRI can provide functional information on whole tumor aggressiveness and intratumoral heterogeneity, which contributes to the characterization of breast tumors (3, 4). Radiomics, radiogenomics and AI studies have been using MRI data to predict breast cancer subtypes, clinical outcomes and response to neoadjuvant chemotherapy (5, 6). The aim of this Research Topic was to discuss the increasing role of breast MRI in improving the management of breast cancer patients, emphasizing technical advances that allow better correlation between MRI findings and histologic, immunohistochemical, and molecular features, as well as response assessment and treatment outcomes.

MRI has proven to be superior to conventional imaging (mammography and ultrasound) for preoperative staging and response evaluation to neoadjuvant chemotherapy (7–10). When used for locoregional staging, MRI is able to identify additional tumor foci in about 20% of patients on the same breast and 5.5% on the contralateral breast, modifying treatment in up to one third of patients with breast cancer (11). Besides that, MRI may improve the characterization of regional lymph node basins (12). In example, Zhou et al. presented a case of a breast cancer patient with intercostal lymph node metastasis identified only on MRI.

The papers published in this special issue studied technical advances that have allowed MRI to evolve in the characterization of breast lesions and artifacts with no need for contrast administration. Li et al. performed a meta-analysis of 13 studies on the differential diagnosis of breast tumors using diffusion kurtosis imaging (DKI)-parameters, including 867 malignant and 460 benign breast lesions. They showed that DKI-derived mean kurtosis (MK) and mean diffusivity (MD) can be used to discriminate breast tumors and to differentiate invasive ductal carcinoma from ductal carcinoma in situ. Prvulovic Bunovic et al. showed that magnetic resonance spectroscopy (MRS) can be used as an additional tool for improving specificity in breast cancer detection. However, although elevation of the choline peak on MRS has a good sensitivity and specificity in breast cancer detection, both are significantly lower than those of multiparametric MRI. Rizzo et al. compared unenhanced MRI (including diffusion-weighted and T2-weighted imaging) combined with digital breast tomosynthesis (DBT) and dynamic contrast enhanced (DCE)-MRI in 84 women with known breast cancer. Despite DCE-MRI was the most sensitive imaging technique in breast cancer preoperative staging, unenhanced MRI combined with DBT demonstrated good sensitivity and accuracy in lesion detection and tumor size assessment and could be a valid alternative tool for preoperative staging. At last, Eskreis-Winkler et al. developed a multispectral imaging (MSI) technique to accurately identify metallic biopsy markers on breast MRI-guided breast biopsy and may eliminate the need for a post-procedure mammogram, thus improving the clinical workflow and eliminating the use of ionizing radiation. Besides that, this technique could be used to better locate metallic markers in the breast and axilla in cases with complete imaging response after neoadjuvant treatment.

Other papers in this issue demonstrated that MRI can also provide information on tumor aggressiveness and response to treatment. Liu et al. prospectively assessed 67 patients with no special type invasive breast carcinoma submitted to preoperative DCE-MRI and showed that quantitative perfusion parameters can correlate with molecular biological expression and molecular subtypes. Zhang et al. associated tumor Oncotype Dx recurrence score with background parenchymal enhancement (BPE) in the contralateral non-tumor breast in 80 breast cancer patients, suggesting that the breast microenvironment may relate to the likelihood of recurrence and magnitude of chemotherapy benefit. Montemezzi et al. found that radiomic features extracted from 3T DCE-MRI consistently improved predictive models of complete response to neoadjuvant chemotherapy.

In conclusion, breast MRI has emerged as an important complementary and noninvasive tool to assess breast cancer, providing information not only related to disease extent but also related to the tumor biology and prognosis. In this context. this Research Topic is a comprehensive collection of illustrative examples of papers that show the potential of breast MRI to improve our knowledge on the behavior of different breast cancer subtypes, which may aid in directing appropriate personalized treatment in the future.

## Author Contributions

All authors contributed to manuscript, read, and approved the submitted version.

## Funding

This work was partially supported by the Vienna Science and Technology Fund (LS19-018, LS20-065).

## Conflict of Interest

The authors declare that the research was conducted in the absence of any commercial or financial relationships that could be construed as a potential conflict of interest.

## Publisher’s Note

All claims expressed in this article are solely those of the authors and do not necessarily represent those of their affiliated organizations, or those of the publisher, the editors and the reviewers. Any product that may be evaluated in this article, or claim that may be made by its manufacturer, is not guaranteed or endorsed by the publisher.

## References

[1] Waks AG Winer EP. Breast Cancer Treatment. JAMA (2019) 321:288. doi: 10.1001/jama.2018.19323 30667503

[2] Harbeck N Penault-Llorca F Cortes J Gnant M Houssami N Poortmans P. Breast Cancer. Nat Rev Dis Prim (2019) 5:66. doi: 10.1038/s41572-019-0111-2 31548545

[3] Mann RM Cho N Moy L. Breast MRI: State of the Art. Radiology (2019) 292:520–36. doi: 10.1148/radiol.2019182947 31361209

[4] Iima M Honda M Sigmund EE Ohno Kishimoto A Kataoka M Togashi K. Diffusion MRI of the Breast: Current Status and Future Directions. J Magn Reson Imaging (2020) 52:70–90. doi: 10.1002/jmri.26908 31520518

[5] Pinker K Chin J Melsaether AN Morris EA Moy L. Precision Medicine and Radiogenomics in Breast Cancer: New Approaches Toward Diagnosis and Treatment. Radiology (2018) 287:732–47. doi: 10.1148/radiol.2018172171 29782246

[6] Bitencourt A Daimiel Naranjo I Lo Gullo R Rossi Saccarelli C Pinker K. AI-Enhanced Breast Imaging: Where Are We and Where Are We Heading? Eur J Radiol (2021) 142:109882. doi: 10.1016/j.ejrad.2021.109882 34392105 PMC8387447

[7] Ray KM Hayward JH Joe BN. Role of MR Imaging for the Locoregional Staging of Breast Cancer. Magn Reson Imaging Clin N Am (2018) 26:191–205. doi: 10.1016/j.mric.2017.12.008 29622125

[8] Kuhl CK Lehman C Bedrosian I. Imaging in Locoregional Management of Breast Cancer. J Clin Oncol (2020) 38:2351–61. doi: 10.1200/JCO.19.03257 PMC7343437 32442068

[9] Fowler AM Mankoff DA Joe BN. Imaging Neoadjuvant Therapy Response in Breast Cancer. Radiology (2017) 285:358–75. doi: 10.1148/radiol.2017170180 29045232

[10] Rauch GM Adrada BE Kuerer HM van la Parra RFD Leung JWT Yang WT. Multimodality Imaging for Evaluating Response to Neoadjuvant Chemotherapy in Breast Cancer. Am J Roentgenol (2017) 208:290–9. doi: 10.2214/AJR.16.17223 27809573

[11] Plana MN Carreira C Muriel A Chiva M Abraira V Emparanza JI. Magnetic Resonance Imaging in the Preoperative Assessment of Patients With Primary Breast Cancer: Systematic Review of Diagnostic Accuracy and Meta-Analysis. Eur Radiol (2012) 22:26–38. doi: 10.1007/s00330-011-2238-8 21847541

[12] Marino MA Avendano D Zapata P Riedl CC Pinker K. Lymph Node Imaging in Patients With Primary Breast Cancer: Concurrent Diagnostic Tools. Oncologist (2020) 25:e231-e242. doi: 10.1634/theoncologist.2019-0427 32043792 PMC7011661

